# Inverted Encoding Models Assay Population-Level Stimulus Representations, Not Single-Unit Neural Tuning

**DOI:** 10.1523/ENEURO.0098-18.2018

**Published:** 2018-06-05

**Authors:** Thomas C. Sprague, Kirsten C. S. Adam, Joshua J. Foster, Masih Rahmati, David W. Sutterer, Vy A. Vo

**Affiliations:** 1 Department of Psychology, New York University, New York, NY 10003; 2 Department of Psychology and Institute for Mind and Biology, University of Chicago, Chicago, IL 60637; 3 Neurosciences Graduate Program, University of California, San Diego, La Jolla, CA 92093

**Keywords:** cognitive vision, computational neuroimaging, fMRI, inverted encoding model

## Significance Statement

Inverted encoding models (IEMs) are a powerful tool for reconstructing population-level stimulus representations from aggregate measurements of neural activity (e.g., fMRI or EEG). In a recent report, Liu et al. (2018) tested whether IEMs can provide information about the underlying tuning of single units. Here, we argue that using stimulus reconstructions to infer properties of single neurons, such as neural tuning bandwidth, is an ill-posed problem with no unambiguous solution. Instead of interpreting results from these methods as evidence about single-unit tuning, we emphasize the utility of these methods for assaying population-level stimulus representations. These can be compared across task conditions to better constrain theories of large-scale neural information processing across experimental manipulations, such as changing sensory input or attention.

## 

Neuroscience methods range astronomically in scale. In some experiments, we record subthreshold membrane potentials in individual neurons, while in others we measure aggregate responses of thousands of neurons at the millimeter scale. A central goal in neuroscience is to bridge insights across all scales to understand the core computations underlying cognition (Churchland and Sejnowski, 1988). However, inferential problems arise when moving across scales: single-unit response properties cannot be inferred from fMRI activation in single voxels, subthreshold membrane potential cannot be inferred from extracellular spike rate, and the state of single ion channels cannot be inferred from intracellular recordings. These are all examples of an inverse problem in which an observation at a larger scale is consistent with an enormous number of possible observations at a smaller scale.

**Figure 1.**
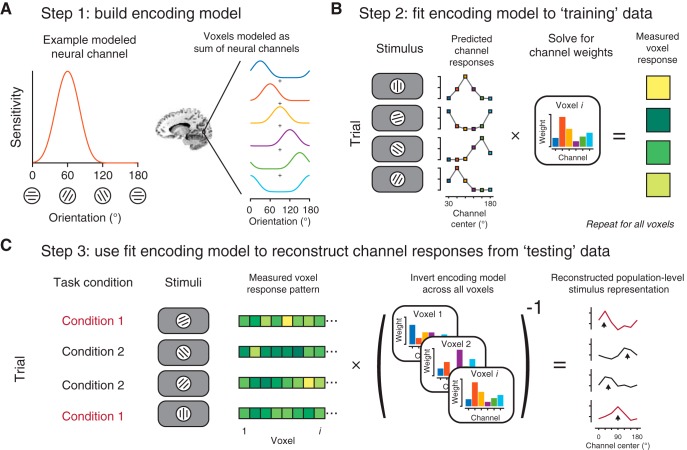
IEM: use neural tuning as an assumption to estimate population-level representations. ***A***, The IEM framework assumes that aggregate neural responses (e.g., voxels) can be modeled as a combination of feature-selective information channels (i.e., orientation-selective neural populations). Tuning properties of modeled information channels are experimenter defined and often based on findings in the single-unit physiology literature. ***B***, Once an encoding model (***A***) is defined, it can be used to predict how each information channel should respond to each stimulus in the experiment. These predicted channel responses are used to fit the encoding model to each voxel’s activation across all trials in a “training” dataset, often balanced across experimental conditions, or derived from a separate “localizer” or “mapping” task. ***C***, By inverting the encoding models estimated across all voxels (typically, within an independently-defined region), new activation patterns can be used to compute the response of each modeled neural information channel. This step transforms activation patterns from measurement space (one number per measurement dimension, e.g., voxel) to information space (one number per modeled information channel, ***A***). These computed channel response functions can be aligned based on the known stimulus feature value on each trial (black arrowheads), and quantified and compared across conditions (e.g., manipulations of stimulus contrast, spatial attention, etc.), especially when a fixed encoding model is used for reconstruction (as schematized here). Cartoon data shown throughout figure.

Recent analytical advances have circumvented challenges inherent in inverse problems by instead transforming aggregate signals from their native “measurement space” (e.g., activation pattern across fMRI voxels) into a model-based “information space” (e.g., activity level of modeled information channels). To make this inference possible, aggregate neural signals (fMRI voxel activation or EEG electrode activity) are modeled as a combination of feature-selective information channels, each with defined sensitivity profiles consistent with the single-unit literature (e.g., experimenter-defined tuning to a particular orientation; Fig. 1*A*; Brouwer and Heeger, 2009, 2011). When an aggregate neural signal is described with such an encoding model, it is possible to invert this model to infer the activity of each channel given a new pattern of neural activity [hence, these methods are often called inverted encoding models (IEMs); Sprague et al., 2015]. Importantly, rather than attempt to solve the inverse problem (how do single-units respond?), this method makes simplifying assumptions that enable transformation of one population-level measurement (aggregate neural signals in voxel or electrode space) into another (stimulus representations in “channel space”). These reconstructed “channel response functions” enable visualization, quantification, and comparison of population-level stimulus representations across manipulations of task conditions (Brouwer and Heeger, 2011, 2013; Scolari et al., 2012; Garcia et al., 2013; Sprague and Serences, 2013; Foster et al., 2017).

Recently, Liu et al. (2018) examined whether an IEM applied to fMRI data can be used to unambiguously infer the underlying response properties of single units. To this end, they manipulated the contrast of orientated gratings, because contrast only affects the amplitude of single-unit orientation tuning functions, but not their tuning width (Sclar and Freeman, 1982). The authors reasoned that, if the width of single-unit tuning functions does not change with stimulus contrast, and if population-level feature reconstructions derived from aggregate neural signals can be used to make meaningful inferences about single-unit tuning, then manipulating contrast should not change the width of population-level channel-response functions.

To test this prediction, the authors used an IEM to reconstruct representations of grating orientations for two different contrast levels. The authors modeled voxel responses as a sum of neural channels tuned to different orientations based on known visual response properties (Fig. 1*A*). After extracting activation patterns from visual cortex, the authors split data from each contrast condition into a training set, used to estimate how each modeled neural channel contributes to each voxel (Fig. 1*B*), and a testing set, which was used in conjunction with the best-fit model from the training set to compute channel response functions (Fig. 1*C*).

The authors found that reconstructed channel response functions in visual cortex were “broader” for low-contrast gratings than for high-contrast gratings (Fig. 2–4; Liu et al., 2018), which they suggest could be interpreted as evidence that single-unit orientation tuning width depends on stimulus contrast. However, because this observation conflicts with demonstrations from single-unit physiology that orientation tuning is contrast-invariant, Liu et al. (2018) sought to resolve this discrepancy using simulations.

The authors simulated cortical fMRI data under different conditions to assess how changes in single-unit responses might be reflected in reconstructed channel response functions. Each simulated voxel’s response was modeled as a noisy weighted sum of orientation-tuned neurons, each with a different orientation preference (Liu et al., 2018, their Fig. 3). Across runs of their simulations, the authors manipulated simulated response properties, like orientation tuning width of constituent model neurons and signal-to-noise ratio (SNR) of the voxel response. The authors found that by decreasing the response amplitude of each simulated neuron (thus, decreasing SNR) without changing the tuning width, they could almost exactly reproduce the broadening in the width of the channel response function when stimulus contrast was decreased (Liu et al., 2018, their Fig. 4). Interestingly, they also found that changes in modeled neural tuning width could alter the width of channel response functions. However, because such broadening is consistent with either a change in SNR or a change in neural tuning width, the authors conclude that it remains impossible to conclusively infer how changes in channel response functions relate to changes in neural tuning. Since it is plausible that low-contrast stimuli evoke weak, noisy responses relative to high-contrast stimuli, the authors argue this is a more parsimonious explanation for their observed data than overturning well-characterized results from the animal physiology literature and inferring that single-unit tuning properties change with contrast. Accordingly, the authors concluded that “changes in channel response functions do not necessarily reflect changes in underlying neural selectivity” (Liu et al., 2018, p 404).

This report makes an important contribution in its dissection of how model-based analysis methods can be sensitive to features of the data that might vary across conditions (e.g., SNR), and clearly demonstrates that changes in population-level channel response functions cannot and should not be used to infer changes in unit-level neural tuning properties. However, we would like to emphasize that this is not the intended purpose of the IEM approach, which is designed to assess population-level stimulus representations. Any inferences made about single-unit tuning from channel response functions are plagued by the same pitfalls encountered when attempting reverse inference about single-unit neural signals from aggregate measurements.

These issues are not unique to the IEM technique. For example, they also complicate interpretation of results from popular voxel receptive field (vRF) techniques. In these experiments, stimuli traverse the entire visual display while experimenters measure fMRI responses. Then, they fit a RF model that best describes how each voxel responds given the visual stimulus (Dumoulin and Wandell, 2008; Wandell and Winawer, 2015). Recent studies have demonstrated that changing task demands (e.g., locus of spatial attention) can change the shape and preferred position of vRFs (Sprague and Serences, 2013; Klein et al., 2014; Kay et al., 2015; Sheremata and Silver, 2015; Vo et al., 2017). While it is tempting to infer that single-neuron RFs change accordingly, it could instead be the case that each neuron maintains a stable RF, but different neurons are subject to different amounts of response gain, altering the voxel-level spatial sensitivity profile measured with these techniques. Moreover, because aggregate measurements like fMRI pool over neurons of different types (excitatory vs inhibitory), selectivity widths (narrow vs broad), and cortical layers (e.g., Layer IV vs Layer II/III), the ability to make inferences about single-unit encoding properties is further limited.

Liu et al. (2018)’s report also highlights that it is important to consider how an encoding model is estimated when comparing channel response functions across conditions. In their work, Liu et al. (2018) estimated separate encoding models for each contrast condition (Fig. 1*B*). But because SNR likely differed between conditions, the observed differences between reconstructions may result from differences in the training sets (i.e., different model fits), or from differences in the testing sets (i.e., different reconstructed activation patterns), or from a combination of the two. More generally, this training scheme can pose a problem for researchers who wish to minimize the effect of known SNR differences between their conditions to study some other variable (e.g., the effect of attention), since it is not possible to unambiguously attribute changes in reconstructed channel response functions to changes in the quality of the model fit or the quality of the representation supported by the population activity pattern, which can both differ between conditions. This problem is roughly akin to reporting a change in a ratio, which can result from changes in the numerator, denominator, or both. One way that others have mitigated this issue is by estimating an encoding model (Fig. 1*B*) using an unbiased (equal numbers of trials from each relevant condition) or neutral (entirely separate task used solely for model estimation) set of data. They then apply that single “fixed” encoding model to test data from multiple stimulus conditions to reconstruct stimulus representations from each condition. This implementation has the advantage that researchers can avoid problems with comparing channel outputs from different IEMs, so the only difference between conditions is the data used for stimulus reconstruction (Fig. 1*C*). We note that even with such a procedure the central result in Liu et al. (2018) could remain true: reconstructions under a fixed encoding model could still broaden with lower contrast. But, as discussed above, this would reflect a change in the quality of the population-level representation rather than provide unambiguous evidence for a change in underlying tuning of individual units. When interpreting results from IEM analyses, it is always critical to consider how the model was estimated.

It would be a mistake to conclude from Liu et al. (2018) that the IEM technique is not useful in the context of its intended purpose: to assay properties of large-scale, population-level neural representations. The quality of these large-scale representations surely depends on myriad factors occurring at the single-unit level. It remains a fascinating question to evaluate how single measurement units, at either the neural or voxel level, change their response properties across visual and task manipulations, but the goal of the IEM approach is to assay the net effect of all these modulations on the superordinate population-level representation. Moreover, few behaviors are guided by single neurons in isolation, and so assaying the joint activity of many neurons, and the resulting population-level representations, is necessary to gain insight into the neural underpinnings of cognition (Jazayeri and Movshon, 2006; Ma et al., 2006; Graf et al., 2011). Indeed, IEMs have been used to assay the time course of covert attention (Foster et al., 2017), understand the consequences of attentional manipulations within working memory (Sprague et al., 2016; Rahmati et al., 2018), evaluate how allocation of attention impacts the representation of irrelevant visual stimuli across the visual field (Sprague and Serences, 2013; Vo et al., 2017; Sprague et al., 2018), and probe the influence of top-down expectations on sensory stimulus representations (Myers et al., 2015; Kok et al., 2017).

We do not believe aggregate neural signals will ever be useful for unambiguously inferring single-unit response properties, including feature tuning. However, we see a bright future for collaborative efforts across labs studying similar questions in different model systems, such as human and macaque. When experiments are well-matched between species, both aggregate measurements in humans and single-unit responses in model systems can be used to inform our understanding of neural coding across different cognitive states. In bridging different levels of analysis, Liu et al. (2018) add to the growing literature using data-driven simulations to better understand the relationship between tuning properties and population-level feature representations (Sprague and Serences, 2013; Kay et al., 2015; Vo et al., 2017). Most importantly, their report underscores the importance of avoiding inferences about signal properties, such as single-unit neural feature tuning, that are fundamentally inaccessible via fMRI or EEG, even when using state-of-the-art acquisition and analysis techniques. We hope that future studies take these issues into account when interpreting findings from model-based analyses applied to aggregate measurement tools like fMRI and EEG. Finally, we remain optimistic that the IEM technique, when applied carefully and interpreted appropriately, will continue to reveal how experimental manipulations impact population-level representations of information.

## Synthesis

Reviewing Editor: Bradley Postle, University of Wisconsin Decisions are customarily a result of the Reviewing Editor and the peer reviewers coming together and discussing their recommendations until a consensus is reached. When revisions are invited, a fact-based synthesis statement explaining their decision and outlining what is needed to prepare a revision will be listed below. The following reviewer(s) agreed to reveal their identity: Edward Ester

Reviewer 1:

This paper comments on a recent report by Liu et al.

Briefly, Liu et al. observed a broadening of orientation-specific representations computed using an inverted encoding model (IEM) when stimuli were rendered with low (relative to high) contrast. This result conflicts with earlier reports of contrast-independent spectral tuning in single unit recordings. Using simulations, Liu et al. show that contrast-dependent changes in stimulus representations could be caused by a broadening of the underlying neural responses (i.e., at the single unit level), decreased SNR, or some combination of both. Insofar as contrast-independent tuning is a well-established property of many orientation-selective neurons in early visual cortical areas, Liu et al. argue that changes in SNR are the most likely source of the contrast-dependent changes they observed and make the broader point that it is difficult (if not impossible) to infer the behavior of an operator (e.g., single unit responses) from an aggregate/population measure that pools information from hundreds, thousands, or millions of those operators.

The authors of this commentary agree with the larger point raised by Liu et al. but also defend the IEM as a useful way of characterizing aggregate signals (e.g., BOLD MRI, multichannel EEG/MEG recordings, etc). However, I doubt Liu et al. would disagree with this characterization. The authors do a good job of discussing Liu et al's findings in the context of the broader literature, but on the whole there's not much here that's novel or compelling. If the authors think that Liu et al. are unfairly maligning the IEM approach or are being too broad in their criticism, then they should come right out and say so and provide specific examples which highlight the power of this method (FWIW I happen to agree with this perspective). It may also be worth pointing out that few (if any) complex behaviors are likely to be mediated by one or a small number of neurons, so characterizing these aggregate signals is a useful and important goal in it's own right. So what if we can't infer neural tuning from these population-based measurements. Was that ever the goal?

Reviewer 2:

This commentary reviewed the Liu et al. (2018) paper on the potential misinterpretation of relating population-level channel responses from Inverted encoding models (IEMs) to single-neuron response properties. Instead of focusing on the link between aggregated voxel- or electrode-level responses and underlying neuronal tuning properties, the authors argue that the problems with interpreting ‘reverse problem’ could occur at multiple neural scales, and that IEMs are still valuable in examining and testing theories on large-scale neural information, especially across different experimental conditions and manipulations.

This commentary is a good addition to the Liu et al. (2018) paper as it reemphasizes the importance of studying population-level neural information while acknowledging the gap between single-unit and population-level responses. I have only minor comments for the authors to consider in a revision.

In the last paragraph of page 4, the authors discuss how the issue raised in the Liu et al. (2018) paper could be averted through careful experimental design. While I agree with the authors that different estimation methods of the IEM could have a significant impact on the patterns of results revealed, I do not think it is appropriate to state that using a separate dataset to train IEM will avoid the SNR issue raised in Liu's paper, because the difference in SNR still exists between the high- and low-contrast conditions in the testing dataset, and whether SNR difference in testing dataset alone will result in broader channel tuning functions is still unknown, unless further simulation evidence is provided.

Furthermore, the authors imply that an IEM trained on a combination of low- and high-contrast data, or on a separate dataset is a ‘stable’ model, compared to IEMs trained on low- and high-contrast data separately. The usage of ‘stable’ here is misleading, because an IEM trained on a combination of low- and high-contrast data or on a separate dataset is not more stable than an IEM trained on low- and high-contrast data separately. The critical difference is that by using the former model, people can avoid problems with comparing channel outputs from different IEMs, which is indeed one possible flaw in the Liu et al. (2018) study.

A minor point:

On line 10 of the last paragraph of page 5, I would suggest using “... they are fundamentally inaccessible via fMRI or EEG, ...” to avoid confusion.

## References

[B1] Brouwer G, Heeger D (2011) Cross-orientation suppression in human visual cortex. J Neurophysiol 106:2108–2119. 10.1152/jn.00540.2011 21775720 PMC3214101

[B2] Brouwer G, Heeger D (2009) Decoding and reconstructing color from responses in human visual cortex. J Neurosci 29:13992–14003. 10.1523/JNEUROSCI.3577-09.2009 19890009 PMC2799419

[B3] Brouwer GJ, Heeger DJ (2013) Categorical clustering of the neural representation of color. J Neurosci 33:15454–15465. 10.1523/JNEUROSCI.2472-13.2013 24068814 PMC3782623

[B4] Churchland PS, Sejnowski TJ (1988) Perspectives on cognitive neuroscience. Science 242:741–745. 3055294 10.1126/science.3055294

[B5] Dumoulin S, Wandell B (2008) Population receptive field estimates in human visual cortex. Neuroimage 39:647–660. 10.1016/j.neuroimage.2007.09.034 17977024 PMC3073038

[B6] Foster JJ, Sutterer DW, Serences JT, Vogel EK, Awh E (2017) Alpha-band oscillations enable spatially and temporally resolved tracking of covert spatial attention. Psychol Sci 28:929–941. 10.1177/0956797617699167 28537480 PMC5675530

[B7] Garcia J, Srinivasan R, Serences J (2013) Near-real-time feature-selective modulations in human cortex. Curr Biol 23:515–522. 10.1016/j.cub.2013.02.013 23477721 PMC3608396

[B8] Graf ABA, Kohn A, Jazayeri M, Movshon JA (2011) Decoding the activity of neuronal populations in macaque primary visual cortex. Nat Neurosci 14:239–245. 10.1038/nn.2733 21217762 PMC3081541

[B9] Jazayeri M, Movshon JA (2006) Optimal representation of sensory information by neural populations. Nat Neurosci 9:690–696. 10.1038/nn1691 16617339

[B10] Kay KN, Weiner KS, Grill-Spector K (2015) Attention reduces spatial uncertainty in human ventral temporal cortex. Curr Biol 25:595–600. 25702580 10.1016/j.cub.2014.12.050 PMC4348205

[B11] Klein BP, Harvey BM, Dumoulin SO (2014) Attraction of position preference by spatial attention throughout human visual cortex. Neuron 84:227–237. 10.1016/j.neuron.2014.08.047 25242220

[B12] Kok P, Mostert P, de Lange FP (2017) Prior expectations induce prestimulus sensory templates. Proc Natl Acad Sci USA 114:10473–10478. 10.1073/pnas.1705652114 28900010 PMC5625909

[B13] Liu T, Cable D, Gardner JL (2018) Inverted encoding models of human population response conflate noise and neural tuning width. J Neurosci 38:398–408. 10.1523/JNEUROSCI.2453-17.2017 29167406 PMC5761616

[B14] Ma WJ, Beck JM, Latham PE, Pouget A (2006) Bayesian inference with probabilistic population codes. Nat Neurosci 9:1432–1438. 10.1038/nn1790 17057707

[B15] Myers NE, Rohenkohl G, Wyart V, Woolrich MW, Nobre AC, Stokes MG (2015) Testing sensory evidence against mnemonic templates. Elife 4:e09000. 10.7554/eLife.09000 26653854 PMC4755744

[B16] Rahmati M, Saber GT, Curtis CE (2018) Population dynamics of early visual cortex during working memory. J Cogn Neurosci 30:219–233. 10.1162/jocn_a_01196 28984524 PMC7406129

[B17] Sclar G, Freeman RD (1982) Orientation selectivity in the cat’s striate cortex is invariant with stimulus contrast. Exp Brain Res 46:457–461. 10.1007/BF00238641 7095050

[B18] Scolari M, Byers A, Serences JT (2012) Optimal deployment of attentional gain during fine discriminations. J Neurosci 32:1–11. 10.1523/JNEUROSCI.5558-11.2012 22649250 PMC3384562

[B19] Sheremata SL, Silver MA (2015) Hemisphere-dependent attentional modulation of human parietal visual field representations. J Neurosci 35:508–517. 10.1523/JNEUROSCI.2378-14.2015 25589746 PMC4293407

[B20] Sprague TC, Serences JT (2013) Attention modulates spatial priority maps in the human occipital, parietal and frontal cortices. Nat Neurosci 16:1879–1887. 10.1038/nn.3574 24212672 PMC3977704

[B21] Sprague TC, Saproo S, Serences JT (2015) Visual attention mitigates information loss in small- and large-scale neural codes. Trends Cogn Sci 19:215–226. 10.1016/j.tics.2015.02.005 25769502 PMC4532299

[B22] Sprague TC, Ester EF, Serences JT (2016) Restoring latent visual working memory representations in human cortex. Neuron 91:694–707. 10.1016/j.neuron.2016.07.006 27497224 PMC4978188

[B23] Sprague TC, Itthipuripat S, Vo VA, Serences JT (2018) Dissociable signatures of visual salience and behavioral relevance across attentional priority maps in human cortex. J Neurophysiol. Advance online publication. Retrieved May 16, 2018 10.1152/jn.00059.2018 PMC6032112 29488841

[B24] Vo VA, Sprague TC, Serences JT (2017) Spatial tuning shifts increase the discriminability and fidelity of population codes in visual cortex. J Neurosci 37:3386–3401. 10.1523/JNEUROSCI.3484-16.2017 28242794 PMC5373124

[B25] Wandell BA, Winawer J (2015) Computational neuroimaging and population receptive fields. Trends Cogn Sci 19:349–357. 10.1016/j.tics.2015.03.009 25850730 PMC4484758

